# Addressing Conservation Needs: Genetic Diversity and Population Ecology of the Endemic Tree *Spondias tuberosa* Arruda

**DOI:** 10.1155/2024/5023974

**Published:** 2024-06-06

**Authors:** Raiane Pereira de Sales, Luan Cavalcanti da Silva, Abidã Gênesis da Silva Neves, Cristiane Gouvêa Fajardo, Luciana Gomes Pinheiro, Fábio de Almeida Vieira

**Affiliations:** Federal University of Rio Grande do Norte, Forestry Engineering, Macaíba, Rio Grande do Norte, Brazil

## Abstract

*Spondias tuberosa* Arruda (Anacardiaceae), popularly known as umbuzeiro or imbuzeiro, is a fruit tree native to the semiarid region of Brazil. The extractive harvesting of its fruits contributes significantly to the economy, generating an annual revenue of approximately $4,2 million. The present study aimed to assess the spatial pattern, allometric variations, fruit measurements, and genetic diversity of trees within a remaining forest of the Caatinga biome, with a focus on intrapopulation analysis. We used intersimple repeated sequence markers and the second-order function density of neighbours to determine the genetic and spatial structure. The density of neighbours was highest within a 10-meter radius. Biometric analyses revealed average fruit lengths of 31.12 mm (±0.22), diameters of 28.68 mm (±0.25), and fresh masses of 15.56 g (±0.33). Diaspores exhibited an average length, diameter, and thickness of 19.27 mm, 13.95 mm, and 11.14 mm, respectively, with a fresh mass of 2.28 g. Notably, the fresh mass demonstrated the highest coefficient of variation. Ten molecular markers were selected, generating 103 highly polymorphic loci (99.03%) with an average informative content of 0.45. Nei's diversity index (0.37) and Shannon's index (0.55) indicated moderate genetic diversity. Furthermore, Bayesian analysis revealed a population structure with two distinct genetic groups. The Infinite Allele and Mutation Step Models suggested a significant historical decline in population size, indicative of a genetic bottleneck. As a result, proactive in situ conservation strategies, including establishing protected natural areas, become essential, considering the socioeconomic significance of the species. Additionally, it is recommended to establish germplasm banks for ex situ conservation and the development of managed cultivation initiatives to reduce the pressure on native populations of *S. tuberosa* caused by extraction.

## 1. Introduction

The Caatinga biome, classified as Seasonally Dry Tropical Forest and Woodlands (SDTFW) [1], covers a vast area of approximately 844,453 km^2^. However, with only 7% designated as protected areas, conserving this ecologically important region is critical [2, 3]. Among the native species of the Caatinga is *Spondias tuberosa* Arruda (Anacardiaceae), commonly known as imbuzeiro or umbuzeiro. Native to the semiarid region of Northeast Brazil [4, 5] *S. tuberosa* fruits are highly appreciated due to their bittersweet flavor and high phenolic compound content, contributing to a diet rich in antioxidants [6, 7]. According to the Brazilian Institute of Geography and Statistics [8], the country's fruit production in 2022 was around 14,200 tons, with the state of Bahia and Minas Gerais being the leading contributor, reaching more than 90% of Brazil's total production [8].

Despite its economic and social importance, *S. tuberosa* faces threats such as reduced natural regeneration and the lack of natural dispersers, leading to population decline and local extinctions [9]. The species is crucial for pollinators in late dry and early wet seasons, supplying nectar and pollen to Meliponini bees. Its flowers are entomophilous and self-incompatible [10, 11]. The presence of *S*. *tuberosa* fruit dispersers, once crucial for the Caatinga region, is now limited to only a few areas within the biome due to the reduced presence of native animals [12]. Therefore, it is crucial to understand spatial patterns at the intrapopulation level to gain insights into the relationship between vegetation dynamics and species characteristics, including the biotic and abiotic factors that can impact inter- and intraspecific competition [13–15]. Spatial distribution analysis reveals how individuals are distributed (clustered or dispersed) within the population [14, 15]. Furthermore, this analysis, combined with biometric studies of fruits and seeds, helps understand the population's reproductive strategies and identify phenotypic variations within and between populations [16].

Understanding the growth patterns of forest species is ecologically important. By comparing traits across individuals, we can identify adaptations that allow them to thrive in different habitats [17]. Allometric analyses, which evaluate how physical or physiological traits change with size, are essential for understanding tree life history [18]. These analyses contribute significantly to knowing the variation in tree morphology across forest types and environmental conditions [19].

Additionally, molecular genetic studies play a pivotal role in discriminating genotypes [20] and elucidating the influence of anthropic activities on population evolution [21]. The genetic diversity of a population can experience changes due to human actions, including fluctuations in population size and shifts in the reproductive system [22, 23]. Molecular studies in population genetics aim to aid in conserving forest species of socioeconomic significance [24]. Furthermore, the reproductive status of the sampled trees, which is crucial for assessing genetic diversity, needs to be evaluated [25]. *S. tuberosa* is an andromonoecious tree that produces inflorescences with a flowering period of two to seven days. Blooms typically occur from September to April, peaking in November before the wet season begins. The blooming of *S. tuberosa* is negatively affected by precipitation [11].

In recent years, molecular markers have become indispensable in genetic research, as they enable the detection of intra- and interpopulation polymorphism [26]. Among the markers, Inter Simple Repeated Sequences (ISSR) stand out. They are dominant markers, requiring no prior knowledge of the genome, provide rapid results, yield vast datasets, and are cost-effective compared to other markers [27–29]. Consequently, this technique has become more accessible and is frequently employed in studies exploring population genetic diversity [30–32].

Therefore, this work aimed to evaluate the spatial patterns, physically characterise the fruits and diaspores, analyse allometric variations, and quantify the genetic diversity within a naturally occurring population of *Spondias tuberosa* in a remnant forest of the Caatinga biome in the Northeast region of Brazil. The following questions were answered: (i) Is there spatial aggregation within the *S. tuberosa* population? (ii) Is there a positive correlation between the biometric characteristics of fruits and diaspores, suggesting the expected rounded shape, and are these variations influenced by water content in the composition? (iii) Are there positive correlations among allometric variations, indicating a direct link to plant growth and establishment? (iv) Is there a correlation between the genetic and geographic distances of *S. tuberosa* individuals? (v) Does a genetic bottleneck occur within the *S*. *tuberosa* population?

## 2. Materials and Methods

### 2.1. Study Site

**Figure 1:**
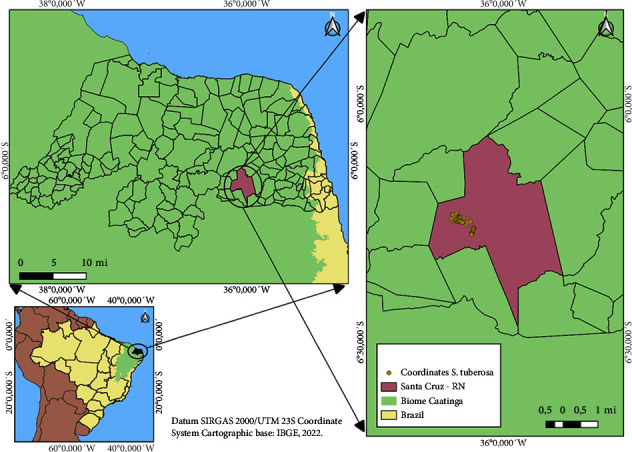
Location map of the study area.

The study was conducted in a natural population at coordinates 6°14′15″ S and 36°06′22″ W in Santa Cruz, Brazil (Figure 1). The predominant vegetation is Hypoxerophilic Caatinga, characterized by a semiarid climate featuring trees and shrubs with thorns [33]. The soil type in this area is Planossolo Solodico, indicating high fertility, sandy texture, and the presence of clay [33]. The population was chosen because it represents one of the few remaining forest refuges in the northern region of the species' distribution. Thus, the *S*. *tuberosa* population in this area is currently considered threatened.

### 2.2. Sampling

#### 2.2.1. Spatial Distribution

A census of all individuals of *Spondias tuberosa* trees (*n* = 53) was conducted within a delimited forest area measuring 270 m^2^. A Garmin GPS device, eTrex®, was used to record the *x* and *y* coordinates of the mature reproductive individuals bearing fruits. The spatial distribution pattern of *S. tuberosa* was determined using the SpPack 1.38 program [34], employing the second-order neighbour density function (NDF). The spatial distribution pattern was assessed across distance classes ranging from 10 m (*t*) to 100 m in correlograms. Simulations were performed to prevent the jagged pattern effect in the correlograms, specifying distance classes with 10 m intervals [13]. Subsequently, the correlograms were generated with NDF statistic values plotted against distance class (*t*) and compared to intervals of complete randomness (confidence interval). These intervals were obtained through 499 replications utilizing the Monte Carlo test (alpha = 0.01) [34].

#### 2.2.2. Allometric Relationships and Biometry of Fruits and Diaspores

For the allometric ratios, measurements were taken for the total height, crown area and circumference at breast height (CBH) in all 53 individuals. These parameters were measured using a tape measure. All CBH values were taken into account according to Scolforo and Mello [35] method: *Ct* = $\sqrt{\left(c1^{2}+c2^{2}+c3^{2}+\ldots c\right)}$, where “*Ct*” is the total circumference and “*ci*” stands for the CBH measurements taken in the field, specifically at 1.30 m above the ground. Subsequently, the “*Ct*” value was converted into the DBH (diameter at breast height), using the formula based on the perimeter of a circle and its diameter: DBH = *Ct*/*π*. The correlations among DBH and crown area, DBH and total height, and crown area and total height were examined.

The biometric assessment of fruits and diaspores was carried out during the natural dispersion period of ripe fruits. A total of 196 ripe fruits were directly collected from the ground near the bole of 23 mother trees bearing fruit at the sampling time. The number of fruits sampled per tree varied according to fruit availability, ranging from 1 to 37, with an average of 8.96 per tree. The sample did not include fruits from trees with questionable ground fruit origins, such as those near other plants. Any fruits found on other trees were deemed immature and were excluded from the collection. Damaged fruits or those displaying signs of predation were also excluded from the analysis.

The collected fruits were carefully stored in separate polyethylene bags, ensuring they were properly labeled with their respective parent trees, and then transported to the laboratory for further analysis. The biometric characteristics were measured using digital callipers to obtain the length (mm), diameter (mm), and thickness (mm), performed. Additionally, an analytical balance was used to measure the fresh mass of the fruits (g). The fruits were pulped manually and washed thoroughly with running water to remove any remaining pulp residue.

The biometric data were submitted to descriptive statistics, including coefficients of variation (CV), skewness (G1) and kurtosis (G2). Deviations from the normal distribution of biometric data were verified using the Lilliefors test (for *K*-samples). The Spearman correlation coefficient (*rs*) was calculated using the BioEstat 5.3 program [36]. We analysed the divergence of biometric characteristics of fruits among trees using the Euclidean distance. The results were then correlated with the Nei genetic distance matrix through the Mantel test using the BioEstat program.

#### 2.2.3. DNA Extraction and ISSR Markers

Leaf tissue fragments were collected from the 23 reproductive trees and placed in plastic tubes containing 2 mL of CTAB 2x solution. We sampled reproductive trees to assess genetic diversity for germplasm banks aimed at conserving the genetic diversity present in mature tree seeds. However, a comprehensive analysis with total sampling is valid. It could be considered in future research for a more comprehensive understanding of genetic diversity within the population. The samples were labeled and transported to the laboratory, where they were stored in a freezer at −20°C until DNA extraction. DNA extraction followed the CTAB method proposed by Doyle [37] with some modifications. Approximately 250 mg of leaf material was combined with a solution containing 100 mM of Tris pH 8.0, 1.4 M NaCl, 20 mM EDTA pH 8.0, 2% (w/v) CTAB, 1% (w/v) PVP-40, and 0.2% (v/v) *β*-mercaptoethanol, preheated to 60°C using a water bath. The extracted DNA was diluted with TE buffer (Tris-HCl 10 mM; EDTA mM pH 8.0) and quantified in an EpochTM spectrophotometer. Subsequently, the DNA was diluted to 50 ng.L^−1^. For polymerase chain reactions (PCR), an initial set of 30 ISSR primers (UBC primer set, primer set #9, University of British Columbia, Vancouver, Canada) were initially employed to conduct amplification tests. The PCR mix consisted of Buffer (10X), BSA (1.0 mg.mL^−1^), MgCl_2_ (50 mM), dNTP (2.5 mM), primer (2 *μ*M), Taq polymerase (U.*μ*L^−1^), DNA (50 ng) and ultra-pure water, with a final volume of 12 *μ*L per sample.

The PCRs were performed in an automatic Biocycler thermal cycler. The samples were denatured at 94°C for 2 min, followed by 37 cycles. Each cycle included a 15 s denaturation at 94°C, followed by 30 s annealing at 47°C, and extension at 72°C for 1 min. A final extension was conducted at 72°C for 1 min, then cooling to 4°C. Following PCR, electrophoresis was performed on a 1.5% (w/v) agarose gel in 1X TAE buffer (Tris-Acetate EDTA) at 100 V for 3 h. A 1,000 base pair molecular weight marker (Ladder) was used to determine the amplified fragments' molecular size. After electrophoresis, the gels were photographed using an ultraviolet light source within the E-Box VX2 Vilbert Lourmat™ equipment. The results of band amplification were used to generate a binary matrix, distinguishing the presence (1) and absence (0) of loci. This matrix was constructed using the Excel 2013 software.

#### 2.2.4. Genetic Analyses

The Polymorphic Information Content (PIC) was calculated to measure the effectiveness of the primers in detecting polymorphisms within each locus of each indicator. The calculation followed the formula proposed by Anderson et al. [38]: PIC_*i*_ = 1 −  Σ_*j*=1_^*n*^*P*_*ij*_^2^, where *P*_*ij*_ is the frequency of the “*j*” allele at the “*i*” marker. The analyses of the genetic diversity parameters were carried out using the POPGENE 1.32 program [39], including the percentage of polymorphic loci (%P), the number of observed alleles (*Na*), the number of effective alleles (*Ne*), Nei's genetic diversity index (*h*), and the Shannon index (*I*).

A dendrogram was constructed using the UPGMA method (Unweighted Pairwise Grouping Using Arithmetic Means), grouping individuals from the population based on their genetic identity as defined by Nei [40]; using the NTSYS program [41]. A correlation analysis was conducted to measure the isolation pattern by distance between Nei's genetic distance and geographic distance (m). This analysis was performed using the Mantel test with 1,000 permutations in the GenAlEx v. 6,502 software [42], implemented within Excel.

A comprehensive Bayesian analysis was conducted to estimate the formation of intrapopulation genetic groups (*K*) using the Structure v.2.2 program [43]. The *K* value ranged from 1 to 4 [44], estimated using the mixed ancestry model (admixture) based on the frequency of correlated alleles. Ten independent runs were performed for each *K* value, comprising 500,000 Monte Carlo simulations via the Markov Chain (MCMC), with a burn-in period of 250,000. The optimal *K* value was determined using the Δ*K* method, implemented in the Structure Harvester program [44, 45].

To identify whether there was a significant reduction in population size, we employed the Bottleneck 1.2.02 software [46]. This analysis is based on allele frequency data and relies on the principle that populations experiencing a genetic bottleneck typically show a decrease in the number of alleles [47]. ISSR markers are best characterized by a mutation model intermediate between the Infinite Allele Model (IAM) [48] and the Stepwise Mutation Model (SMM) [49]. Thus, the IAM and SMM were used to identify potential bottlenecks in this study. The sign test (*α* = 0.05) was then used to analyse allele frequencies to identify significant recent genetic bottlenecks [46].

## 3. Results

### 3.1. Spatial Distribution

**Figure 2:**
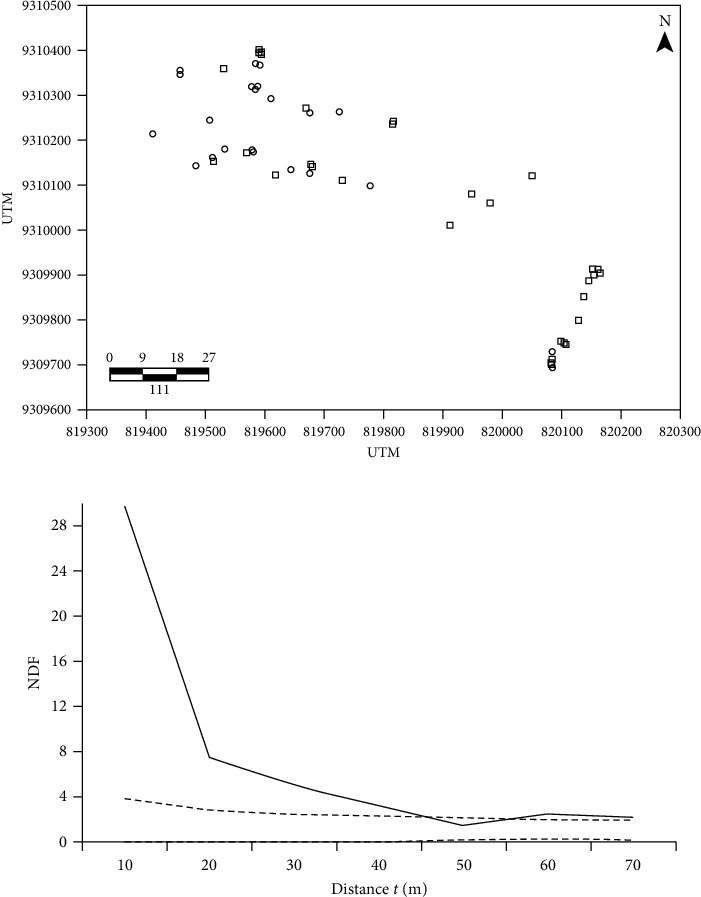
Spatial distribution of *Spondias tuberosa* trees (a). Circles represent trees with fruits (*n* = 23), while squares represent trees without fruit (*n* = 30). The *x-* and *y*-axis are presented in UTM (universal transverse mercator). Spatial pattern obtained through univariate neighbour density analysis (NDF, solid line) of *Spondias tuberosa* trees (b). The dotted line indicates the 99% confidence interval (CI) for the null hypothesis of a completely random spatial pattern.

A total of 53 *S. tuberosa* trees were identified within the study area (Figure 2(a)). The spatial pattern of *S. tuberosa* population indicated a higher density of neighbours (NDF) within a 10-meter radius, indicating an aggregated spatial pattern. This pattern remained significant up to a radius of 55 m from the individual, with NDF values close to those expected under complete randomness (Figure 2(b)).

### 3.2. Allometric Relationships and Biometry of Fruits and Diaspores

Significant allometric correlations were observed between DBH, total height, and canopy area (Table 1).

Fruits had average lengths of 31.12 mm, diameters of 28.68 mm, and fresh masses of 15.56 g (Table 2). Additionally, diaspores exhibited an average length of 19.27 mm, diameter of 13.95 mm, thickness of 11.14 mm, and fresh weight of 2.28 g (Table 2).

Analysis of biometric variables revealed significant correlations. Among fruits, the strongest correlation was observed between fresh mass and pulp yield (*rs* = 0.99). Similarly, for diaspores, the highest correlation was found between fresh mass and diaspore thickness (Table 3).

#### 3.2.1. Genetic Diversity

From the initial set of 30 primers, ten were chosen based on informative criteria: number of bands, clear visualization pattern, and resolution. These ten primers amplified a total of 103 loci (Table 4). The number of loci amplified by each primer ranged from seven to twelve, with an average of 10.3 loci per primer (Table 4). The percentage of polymorphic loci was 99.03%.

#### 3.2.2. Genetic Structure

The average number of observed alleles (Na) was 1.99 (±0.10, standard deviation, s.d.), with the number of effective alleles (Ne) equal to 1.65 (±0.28, s.d.). The Nei index (*h*) was 0.37 (0.12, s.d.). The Shannon index (*I*), which can range from 0 to 1 (with 1 representing the highest population diversity), was observed to be 0.55 (±0.14, s.d.) for the studied population.

The Mantel test showed a positive correlation between genetic and geographic distances (*R*^2^ = 0.096, *y* = 0.0005*x* + 0.5336, *P* < 0.003). The correlation between the Euclidean distance matrix and the genetic distance matrix indicates a relationship between the evaluated biometric characteristics and the genetic distance among *S. tuberosa* trees, with a correlation coefficient (*r*) of 0.16 (*P*=0.01).

**Figure 3:**
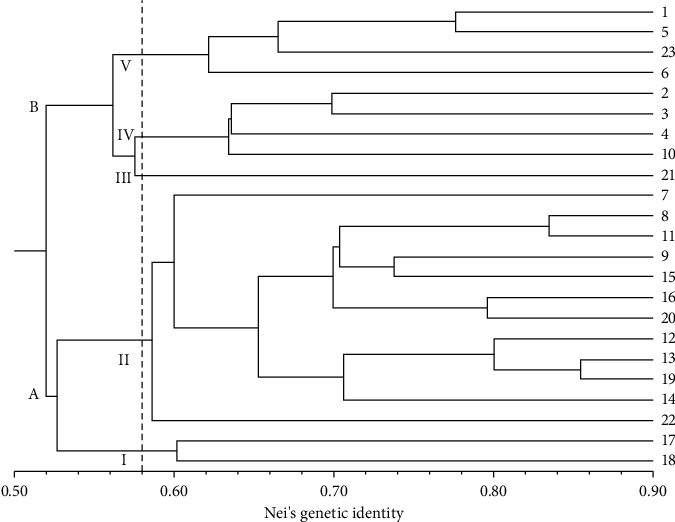
UPGMA dendrogram based on Nei's genetic identity among individuals within the *Spondias tuberosa* population (*n* = 23). The dashed line delimits the groups that clustered at a value of 0.58.

The dendrogram (Figure 3) revealed two main groups (A and B) representing distinct genotypic clusters. These clusters were further divided to identify individuals suitable for germplasm banks for ex situ conservation. Group A contained two subgroups (I and II), while Group B had three (III, IV, and V). Notably, individuals 13–19 exhibited the highest degree of similarity, within a large cluster denoted as G-II. This cluster comprises twelve individuals with greater genetic similarity to each other. In total, five similarity groups (G) were identified based on shared genotypes: G-I (individuals 18 and 17), G-II (22, 14, 19, 13, 12, 20, 16, 15, 9, 11, 8, and 7), G-III (21), G-IV (10, 4, 3, and 2), and G-V (6, 23, 5, and 1). Individual 21 (G-III) showed the highest divergence within these groups. The cophenetic correlation was 0.87.

**Figure 4:**
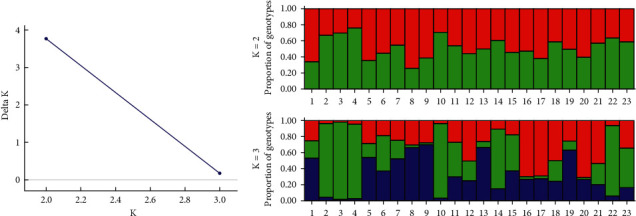
Bayesian analysis-derived *K* value using the Δ*K* method, indicating the number of genetic groups within *Spondias tuberosa* trees (a). Genetic clusters among the twenty-three individuals of *Spondias tuberosa* (b), delineated by vertical bars, with distinct colors representing different groupings (*K* = 2 and *K* = 3).

The Bayesian analysis verified two distinct genetic groups (*K* = 2), as indicated by the Δ*K* values (Figure 4(a)). Consequently, the 23 individuals studied are categorized into two genetic clusters, according to their proportion of genotypes (Figure 4(b)).

#### 3.2.3. Population Decline (Genetic Bottleneck)

Using the Infinite Allele (IAM) and (Step Mutation) SMM models, equilibrium tests were conducted to assess the balance between mutation and genetic drift within the population. The results suggest a population decline, with a *P* value of <0.04. This decline is supported by the discrepancy between the expected (*n*) and observed (He) number of loci with excess heterozygosity for both models. Specifically, the IAM revealed an expected (*n*) of 24 loci compared to the observed (He) of 78 loci (*P* < 0.00). Similarly, the SMM exhibited an expected (*n*) of 29 loci versus an observed (He) of 73 loci (*P* < 0.04).

## 4. Discussion

The spatial pattern may result from different processes, including seed dispersal, intra- and interspecific interactions, and site heterogeneity [14]. These factors collectively create diverse growth conditions, potentially influenced by topography and nutrient availability [15, 50]. Our study suggests that *S. tuberosa* trees exhibited an aggregated distribution, with a higher density occurring within 10 meters of each other. This suggests that a significant proportion of seeds are deposited close to the mother plant, potentially facilitated by zoochoric dispersion [9]. The primary natural dispersers of *S. tuberosa* fruits include brown deer (*Mazama gouazoubira*), agouti (*Dasyprocta cf. prymnolopha*), peccary (*Tayassu tajacu*), fox (*Dusicyon thous*), tegu (*Tupinambis merianae*), and short armadillo (*Euphractus sexcinctus*) [9]. However, these dispersers were once widespread in the Caatinga but are now limited to only a few regions in the biome. This has contributed to a significant decline in the *S. tuberosa* populations in the Caatinga. Our finding that *S. tuberosa* exhibits an aggregated distribution aligns with other studies [51, 52]. Understanding these spatial patterns provides valuable insights into species behaviour and can inform conservation efforts in the Caatinga region.

Our results indicated that diameter at breast height (DBH) is positively correlated with both tree height and canopy area in *S. tuberosa*. This confirms the importance of DBH as a key variable for growth in this species. It is important to consider that environmental factors, such as light exposure and nutrient availability, can also influence variations in these allometric relationships among trees [53, 54]. Overall, studies on allometric relationships are essential for understanding plant biomechanics, ecology, and evolution [55]. Allometric relationships offer a valuable tool for understanding how plants allocate resources. These relationships shed light on biomass measurement, carbon content estimation, and supporting forest restoration and species conservation efforts [55–59]. By understanding how different parts of a *Spondias tuberosa* tree grow in relation to its size, we can further explore this approach in this specific context.

The biometric analysis of *S. tuberosa* fruits revealed that individual trees (7, 8, 9, 10, 11, 18, 19, 20, 23, 24, 25, 28, 30, 31, 35, and 50) produced fruits with remarkable characteristics, including length, diameter, and fresh mass. These trees may have the potential for increased fruit production, both for commercial markets and direct consumption (*in natura*). The analysis also showed lower variability in fruit length and diameter compared to other characteristics. As with many tropical trees, environmental factors such as water and nutrient availability play a significant role in fruit development, especially for fleshy fruits like *S. tuberosa* [60]. Our study of biometric characteristics, including fresh mass, length, and width, revealed values similar to those reported in previous studies [61, 62]. The positive correlation between fresh mass and fruit pulp yield suggests that fruits with higher fresh mass likely contain more pulp. This finding has significant implications for managing *S. tuberosa* populations and fruit extraction. By selecting trees that produce fruits with higher fresh mass, we can efficiently collect fruits ideal for the consumer market while using seeds for cultivating productive forests. These practices can improve the income of communities that rely on *S. tuberosa* extraction, while simultaneously contributing to the conservation of the species in its natural habitat.

The selected loci were moderately informative, with a polymorphic information content ranging from 0.41 to 0.49. This indicates that the primers used in this study were effective in detecting genetic variation among *S. tuberosa* trees. The Mantel test indicated a positive correlation between the evaluated fruit characteristics and the genetic distance among trees. This suggests that variations in fruit characteristics of *S. tuberosa* are influenced not only by environmental factors but also by the genetic makeup of the trees.

The average Shannon index obtained (0.37) is slightly higher than the values reported for other species with similar life histories to *S. tuberosa* [63]. These similarities include perennial long-duration life habits, regional geographic distribution, cross-pollination mating systems, zoochoric seed dispersal, and late successional stage. In Brazil's tropical forest, most zoochoric species have juicy fruits, and their seeds are typically dispersed by frugivorous vertebrates [64]. It is important to note that the *S. tuberosa* relies exclusively on zoochory for seed dispersal [9].

The Nei's genetic diversity and Shannon's index revealed values similar to those reported in previous studies on *Spondias* spp. that employed ISSR markers [65, 66]. These results suggest relatively low genetic variation within the *S. tuberosa* population studied. Although the Mantel test indicated a weak positive correlation between the genetic and geographic distance, both Bayesian and UPGMA analyses identified similar genetic groups. These groups can be useful for selecting genetically diverse individuals for germplasm banks. Such banks can be established for ex situ conservation of genetic diversity by collecting seeds from mature trees. The Mantel test showed a positive correlation between biometric traits (fruit characteristics) and the genetic distances estimated using ISSR markers. Previous studies have also observed significant correlations between morphological traits and molecular markers [67, 68]. Phenotypic traits, like fruit characteristics, offer practical advantages for easy identification and can be used as a comprehensive approach to assess genetic relatedness among different germplasm [28]. This approach could contribute to future plant breeding and improvement program collections [68].

Our findings suggest a genetic bottleneck in the studied *S. tuberosa* population, indicated by the reduced number of genotypes. Anthropogenic activities likely play a significant role in reducing the *S. tuberosa* population, such as the observed infrastructure expansion for recreational and creation of new tourist trails within the study area. The detection of genetic bottlenecks is crucial as it can signal threats to the long-term survival of a species [69]. The IAM and SMM models are consistent with the low levels of genetic diversity found in this study. In small and isolated populations, genetic drift can lead to deviations from the expected balance of gene frequencies in a short period, causing a loss of allele. Over time, this process can intensify, resulting in increased inbreeding among plants [70].

The growing commercial demand for *S. tuberosa* fruits necessitates the development of comprehensive conservation strategies [66]. One effective approach to preserving *S. tuberosa* populations is implementing local management practices, such as rotating fruit collections during harvests and leaving open and cracked fruits on the ground [65]. Through non-deforestation and cutting in collection areas, the maintenance of trees plays a vital role in preserving tree dynamics and the genetic diversity of managed *S. tuberosa* populations. In this way, it is possible to support continuous plant regeneration without reducing genetic diversity. This, in turn, facilitates sustainable fruit consumption and commercialization. Additionally, education programs within the tourist enterprises and surrounding communities are essential to ensure the conservation of *S. tuberosa*, recognizing its cultural and economic significance. Establish germplasm banks for ex situ conservation of genetic diversity is another valuable recommendation.

## 5. Conclusion

Our study aimed to comprehensively analyse a *S. tuberosa* population within a Caatinga biome forest, focusing on intrapopulation characteristics. We investigated the spatial distribution pattern, allometric relationships among tree structures, fruit size and pulp content, and most importantly, the genetic diversity of the population. Key findings revealed an aggregated spatial distribution, suggesting limited seed dispersal from the mother tree. Allometric analysis showed a positive correlation between diameter at breast height (DBH) and both tree height and canopy area, indicating a growth that enhances resistance to external damage. Additionally, a positive correlation between fruit size and pulp mass allows consumers to select fruits with higher pulp content. However, a critical finding was the low genetic diversity and evidence of genetic bottlenecks within the population.

These findings highlight the need for immediate conservation action to ensure the long-term sustainability of *S. tuberosa* in the Caatinga biome. Implementing a multifaceted conservation strategy is crucial. This strategy should encompass in situ conservation efforts, community engagement for sustainable management plan development, education programs, promotion of sound harvesting practices, and reforestation initiatives in disturbed areas. These combined actions can significantly contribute to the preservation of *S. tuberosa* for future generations [71].

## Figures and Tables

**Table 1:** Spearman correlation analysis for allometric data from a population of *Spondias tuberosa* (*n* = 53).

Relationship	Spearman correlation	
	*rs*	*P*
DBH × total height	0.51	<0.01
DBH × canopy area	0.53	<0.01
Total height × canopy area	0.51	<0.01

*rs*: Spearman correlation, *P*: significance level.

**Table 2:** Descriptive statistics for biometric evaluation of *Spondias tuberosa* fruits and diaspores from 23 trees.

Biometric characteristics	*n*	Max.	Min.	Mean ± standard error	CV (%)	G1	G2
*Fruits*							
Length (mm)	196	39.02	20.05	31.12 ± 0.22	10.05	−0.31	0.25
Diameter (mm)	196	35.48	17.73	28.68 ± 0.25	12.03	−0.88	0.49
Fresh mass (g)	196	26.47	4.11	15.56 ± 0.33	29.69	−0.56	−0.06

*Diaspores*							
Length (mm)	196	25.03	15.07	19.27 ± 0.13	9.08	0.23	−0.14
Diameter (mm)	196	18.56	10.32	13.95 ± 0.09	9.39	0.36	0.71
Thickness (mm)	196	14.64	8.06	11.14 ± 0.09	10.79	0.51	0.41
Fresh mass (g)	196	8.90	0.87	2.28 ± 0.06	34.05	0.67	1.37

*n*: sample size, Max: maximum, Min: minimum, CV: coefficient of variation, G1: asymmetry, G2: kurtosis.

**Table 3:** Spearman correlation analysis for biometric data in *Spondias tuberosa* population (*n* = 23).

Correlations	*rs*
*Fruits*	
Fresh mass × fruit length	0.89^*∗*^
Fresh mass × fruit diameter	0.92^*∗*^
Fruit mass × fruit diameter	0.77^*∗*^
Fresh mass × pulp yield	0.99^*∗*^

*Diaspores*	
Fresh mass × diaspore thickness	0.81^*∗*^
Fresh mass × diaspore length	0.64^*∗*^
Fresh mass × diaspore diameter	0.79^*∗*^
Diaspore diameter × diaspore thickness	0.78^*∗*^
Diaspore length × diaspore diameter	0.48^*∗*^
Diaspore length × diaspore thickness	0.36^*∗*^
Fresh mass × pulp yield	0.63^*∗*^

^*∗*^ = *P* < 0.05.

**Table 4:** Nucleotide sequence of ISSR primers, number of loci, and PIC values for each primer.

ISSR primers	Sequence (5′-3′)	Number of loci	PIC
M1	CAAGAGAGAGAGA	7	0.49
UBC 807	AGAGAGAGAGAGAGAGT	10	0.48
UBC 808	AGAGAGAGAGAGAGAGC	10	0.46
UBC 813	CTCTCTCTCTCTCTCTT	9	0.49
UBC 818	CACACACACACACACAG	11	0.42
UBC 825	ACACACACACACACACT	12	0.42
UBC 826	ACACACACACACACACC	12	0.46
UBC 841	GAGAGAGAGAGAGAGAYC	11	0.43
UBC 862	AGCAGCAGCAGCAGCAGC	12	0.41
UBC 873	GACAGACAGACAGACA	9	0.47

	Average	10.3	0.45

*Y* = pyrimidine (C our T); PIC = polymorphic information content.

## Data Availability

The datasets produced and/or examined during the present investigation are freely available within the manuscript and can be obtained from the corresponding author upon reasonable request.
